# The Influence of Molecular Weight and Comonomer on the Shear Creep of Polyethylene

**DOI:** 10.3390/polym18080926

**Published:** 2026-04-10

**Authors:** Jingwen Li, Zhilan Jin, Yanshu Wang, Shicheng Zhao, Chunlin Ye

**Affiliations:** 1 Shanghai Key Laboratory of Multiphase Materials Chemical Engineering, School of Chemical Engineering, East China University of Science and Technology, Shanghai 200237, China; 2 State Key Laboratory of Polyolefins and Catalysis, Shanghai 200062, China; 3 Shanghai Key Laboratory of Catalysis Technology for Polyolefins, Shanghai 200062, China

**Keywords:** shear creep, polyethylene, dynamic mechanics analysis, rheology, entanglement density

## Abstract

The occurrence of shear creep in polyethylene under applied stress results in deformation, which restricts the service life of the final product. However, the factors influencing shear creep and its underlying mechanisms remain unclear. This article investigates the effects of average molecular weight and comonomer on the shear creep behavior and underlying mechanisms of high-density polyethylene (HDPE). The materials chosen were HDPE with weight-average molecular weights (Mw) of 148,100, 191,800, 226,500, 252,700 and 325,100 g/mol, as well as copolymers incorporating propylene or octene as comonomers. The results indicate that creep deformation decreases with increasing Mw, and that polyethylene copolymers incorporating propylene and octene cause increased creep deformation compared to homopolymers. Dynamic mechanical analysis (DMA) and rheological testing were used to investigate the influence of Mw and comonomer on shear creep behavior. The experimental results demonstrate that increasing the weight-average molecular weight enhances molecular chain entanglement, thereby improving creep resistance. The incorporation of comonomers introduces branches into the polyethylene structure, reducing entanglement density and leading to diminished creep resistance. This study provides valuable insights and references for the development of polyethylene materials that resist shear creep.

## 1. Introduction

Polyethylene has attracted much attention in industry and academia due to its excellent properties, including high wear resistance, corrosion resistance, and impact resistance [1,2]. It is widely used in medical materials, packaging materials, building materials, and other fields [3]. However, polyethylene is susceptible to deformation, primarily manifested through its viscoelastic behavior. Under sustained external loads, polyethylene undergoes creep, which greatly limits its applicability in various engineering and industrial fields [4,5].

The significance of this issue is widely recognized, leading to considerable research efforts focused on the creep behavior of polyethylene. Creep is a fundamental property of viscoelastic materials [6,7], which refers to the behavior in which the deformation of polymer materials gradually increases over time under a certain temperature and a constant external force [8,9,10]. The process of polymer creep can be divided into three stages: (1) deceleration creep stage, (2) steady-state creep stage, and (3) acceleration creep stage [11,12,13].

Under external mechanical loads such as tensile, bending, shear, and compressive stresses, polyethylene exhibits different creep behaviors. It is well established from numerous studies that molecular weight and comonomer content are critical factors affecting the tensile creep behavior of polyethylene [14,15]. In practical applications, polyethylene is rarely subjected only to uniaxial tensile stress; in many service environments, it experiences creep under shear stress. Therefore, the study of shear creep behavior is equally important for understanding the long-term performance of the material. M. H. Wagner et al. [16] conducted constant shear stress creep experiments on LDPE and developed a constitutive model capable of accurately predicting its creep behavior. Claus Gabriel et al. [17] investigated the creep response of two metallocene-based linear low-density polyethylene (LLDPE) materials and found that the branched polymer exhibited greater deformation and higher creep compliance under identical conditions. Pawan K. Agarwal [18] examined the shear creep and recovery behavior of three low-density polyethylene (LDPE) variants, revealing that molecular weight distribution significantly influences shear creep performance, with samples exhibiting narrow distributions demonstrating enhanced creep resistance. In addition, there have been other studies on the influence of microstructure on material properties. Fazeli N et al. [19] conducted research on ethylene/1-butene copolymers and found that reaching a certain number of branches reduces the flow resistance between chains. Chen B et al. [20] investigated cross-linked networks and found that stress relaxation time typically decreases with decreasing cross-linking density. Simanke A G et al. [21] investigated the effect of crystallinity on the stress–strain behavior of ethylene/α-olefin copolymers and found that higher crystallinity led to increased yield stress and stress resistance under a tensile force field. Despite these valuable insights, there is still a lack of systematic research and specifically an understanding of the shear creep behavior of high-density polyethylene (HDPE). In addition, the potential mechanisms by which molecular weight and comonomers govern the shear creep process of HDPE have not been fully elucidated.

In this article, the effects of molecular weight and comonomer on the shear creep behavior and underlying mechanisms of HDPE were systematically investigated. The selected materials include polyethylene with weight-average molecular weights (M_w_) of 148,100, 191,800, 226,500, 252,700 and 325,100 g/mol, as well as copolymers incorporating propylene or octene as comonomers. The influence of molecular weight and comonomer type on the shear creep behavior of HDPE was first investigated. Following this, dynamic mechanical analysis (DMA) and rheological tests were employed to characterize the chain entanglement density and relaxation time, thereby elucidating the underlying influence mechanism. This study provides a theoretical basis for developing polyethylene that is more resistant to creep.

## 2. Experimental

### 2.1. Materials

In this study, the polyethylene materials were synthesized in our laboratory.

The materials are abbreviated as PE14, PE19, PE22, PE25, PE32, PE14-propylene and PE14-octene, with the number representing the weight-average molecular weight. The specific information of the materials is shown in Table 1. PE14, PE19, PE22, PE25 and PE32 are homopolymers of polyethylene. PE14-propylene is an ethylene-propylene copolymer. PE14-octene is an ethylene–octene copolymer.

### 2.2. Testing and Characterization

#### 2.2.1. Sample Preparation

For the shear creep test, circular samples with a diameter of 28 mm and a thickness of 2 mm were prepared by a micro-injection molding machine (Mini Jet Pro, Thermo Fisher Scientific, Braunschweig, Germany). The experiment set the melting temperature to 270 °C, the injection pressure to 650 kPa for 15 s, the holding pressure to 550 kPa for 30 s, and the mold temperature to 50 °C.

For the dynamic mechanics analysis test, splines measuring 2 mm × 8 mm × 20 mm were prepared by a micro-injection molding machine. The experiment set the melting temperature to 270 °C, the injection pressure to 650 kPa for 15 s, the holding pressure to 550 kPa for 30 s, and the mold temperature to 50 °C.

To ensure comparability, the rheological samples were prepared following the same procedure as for the shear creep test.

#### 2.2.2. Melting Performance Test

The differential scanning calorimetry (DSC3+, Mettler Toledo, Greifensee, Switzerland) was used to test the melting behavior of all samples. To eliminate thermal history, the samples were first held at 200 °C for 10 min. Subsequently, they were cooled from 200 °C to 25 °C at a rate of 20 °C/min. Following this, the samples were reheated from 25 °C to 200 °C at the same rate to obtain the subsequent heating–melting curves. Melting parameters were derived from these thermograms.

#### 2.2.3. Shear Creep Test

The rotational rheometer (MCR-302, Anton Paar, Shanghai, China) was used to test the creep behavior of all samples. Shear creep experiments were conducted under a constant stress of 200 Pa, both above and below the melting point of the sample. A parallel plate rotor (PP25) was selected for the measurements. Then the shear stress was removed to continue the experiment. During the experiment, points were taken according to the logarithmic rule, 185 data points were recorded for creep deformation during shear stress, and 373 data points were recorded for creep recovery.

#### 2.2.4. Dynamic Mechanics Analysis

The glass transition temperature and plateau modulus of the molecular chain were determined using a dynamic thermomechanical analyzer (DMA1, Mettler Toledo, Switzerland). The sample size was 10 mm × 8 mm × 2 mm in tensile mode, with a frequency of 1 Hz, a temperature range of −160 °C~200 °C, and a heating rate of 10 °C/min.

#### 2.2.5. Rheological Test

The rotational rheometer was used to test the rheological properties. The frequency sweep tests were conducted using a flat plate equipped with the PP08 rotor. The strain was set at 1%, and the angular frequency was swept from 0.01 to 100 rad/s at a test temperature of 210 °C.

## 3. Results and Discussion

### 3.1. Shear Creep Behavior of Polyethylene with Different Molecular Weights

**Figure 1:**
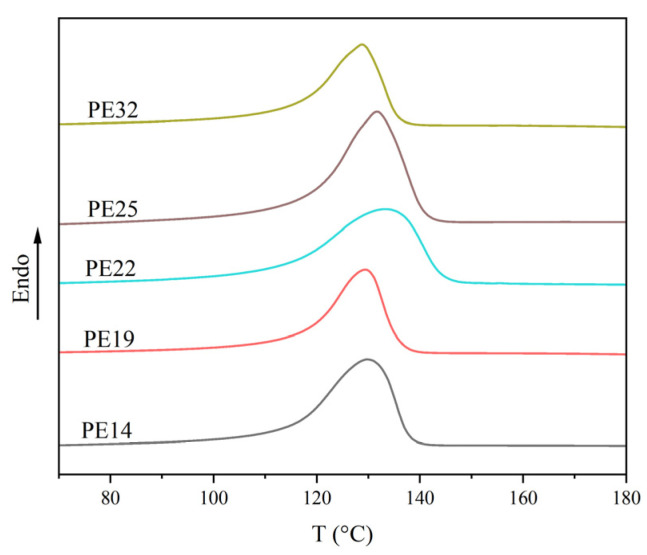
DSC heating–melting curves of polyethylene with different molecular weights.

To investigate the effect of weight-average molecular weights (M_w_) on shear creep, polyethylene homopolymer samples PE14, PE19, PE22, PE25 and PE32 with different M_w_ were selected for testing. To determine the appropriate temperatures for subsequent creep tests, the melting behavior of the samples with different M_w_ was examined. The melting curves for the five samples are presented in Figure 1.

Based on the melting parameters of the samples, two experimental temperatures were selected: 90 °C (below the T_m_) and 210 °C (above the T_m_). This temperature selection was intended to ensure good resolution and low measurement error in the creep behavior within the low-temperature region while preventing thermal decomposition of the samples in the high-temperature region.

**Figure 2:**
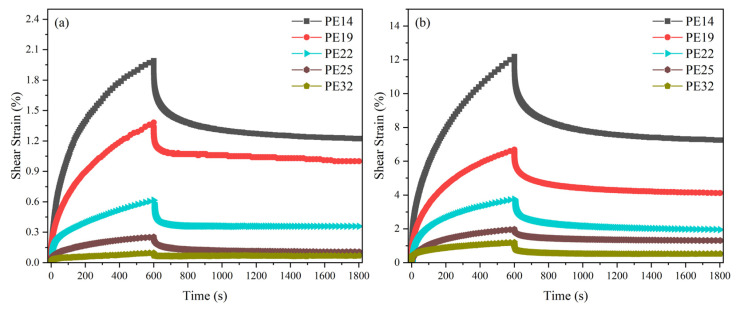
Shear creep and creep recovery curves of PE14, PE19, PE22, PE25 and PE32 under (**a**) 90 °C and (**b**) 210 °C.

The shear creep test results at two selected temperatures are shown in Figure 2.

As shown in Figure 2, during the shear creep process at two temperatures, all samples underwent a transition from the deceleration creep stage to the steady-state creep stage. In the deceleration creep stage, the strain rate rapidly reached its maximum value immediately after load application and then gradually decreased with time. In the steady-state creep stage, the strain rate stabilized and remained relatively constant. At 90 °C, compared with the initial state, the maximum shear strains of PE14, PE19, PE22, PE25, and PE32 were 2.0%, 1.4%, 0.6%, 0.3%, and 0.1%, respectively (Figure 2a). Compared with the initial state at 210 °C, the maximum shear strains of PE14, PE19, PE22, PE25, and PE32 were 12.2%, 6.7%, 3.8%, 2.0%, and 1.2%, respectively (Figure 2b). In the experiments at both temperatures, PE14 exhibited the highest creep strain, whereas PE32 showed the lowest, meaning that the maximum creep strain decreased with increasing molecular weight. Due to the negative correlation between creep strain and creep resistance, these results indicate that PE32 has the best shear creep resistance performance among the tested materials. This trend demonstrates that for these ethylene homopolymer samples, creep resistance improves with increasing M_w_. At two experimental temperatures below and above T_m_, the order of creep resistance among polyethylenes with different M_w_ remained consistent, indicating that the amorphous region dominates the shear creep behavior. In terms of creep recovery behavior, all samples underwent recovery at both temperatures, characterized by both instantaneous and delayed recovery. Notably, PE14 exhibited the highest residual permanent deformation, while PE32 showed the lowest.

### 3.2. Analysis of Shear Creep Mechanism of Polyethylene with Different Molecular Weight

**Figure 3:**
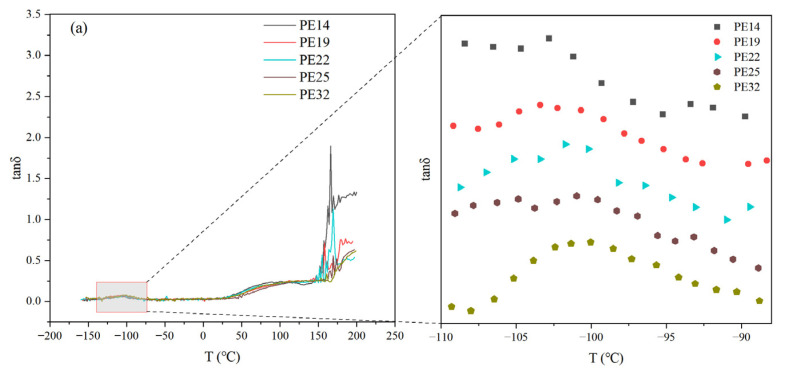

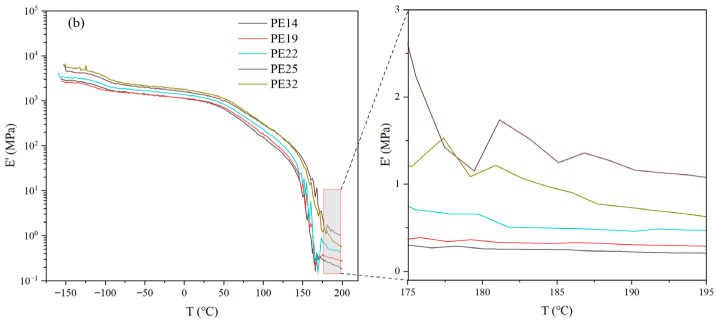
(**a**) The tan δ and (**b**) the storage modulus E’ change-with-temperature curves of PE14, PE19, PE22, PE25 and PE32.

In order to explain the influence mechanism of M_w_ on the shear creep behavior of polyethylene, dynamic mechanical analysis (DMA) was performed on five selected samples. Figure 3a,b show the change curves of the loss factor tanδ and the storage modulus (E’) with temperature at fixed frequency for PE14, PE19, PE22, PE25 and PE32, respectively.

The glass transition temperature (T_g_) is the temperature at which an amorphous polymer transitions from a glassy state to a highly elastic state. T_g_ directly influences the flexibility of polymer chains—generally, a lower T_g_ corresponds to greater material flexibility. The flexibility of these molecular chains is an important factor governing their shear creep behavior. In this analysis, the temperature corresponding to the tanδ peak was selected as the glass transition temperature T_g_ of the sample [22]. According to Figure 3a, the values of T_g_ of the five samples have been compiled in Table 2. The T_g_ values of PE14, PE19, PE22, PE25, and PE32 samples are −103.0 °C, −102.2 °C, −101.3 °C, −100.7 °C and −100.1 °C, respectively.

**Figure 4:**
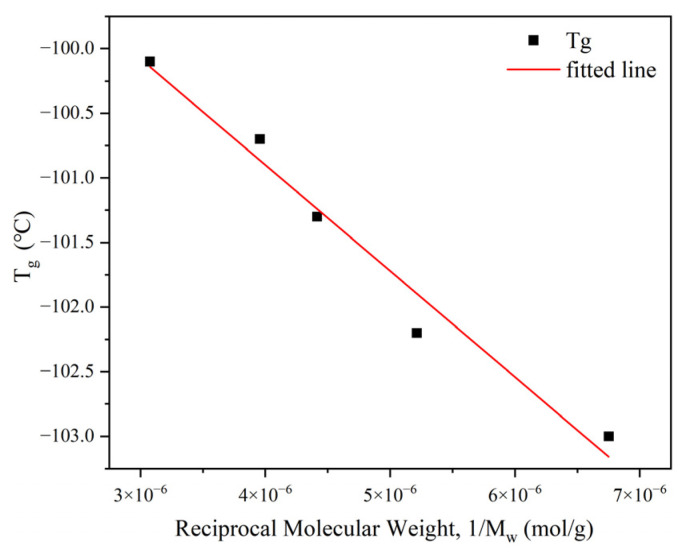
Linear relationship between the plot of T_g_ and reciprocal of the molecular weight, M_w_^−1^.

In the case of linear polymers, T_g_ exhibits a positive dependence on molar mass, varying linearly with the reciprocal of the molecular weight [23]. This phenomenon is attributed to the effect of the movement of molecular end chains. To verify the reliability of the T_g_ test results for polyethylene with different molecular weights, the Fox–Flory equation [23] was used to fit T_g_ and the reciprocal of the molecular weight (Figure 4). The R^2^ value for the fitted data exceeded 0.97, demonstrating an excellent goodness of fit and a highly significant linear relationship. This confirms that the temperature corresponding to the tanδ peak accurately reflected the Tg of the sample.

Among the five samples, PE32 exhibits the lowest T_g_, whereas PE14 shows the highest. A lower T_g_ indicates greater material flexibility and lower internal rotation resistance of its molecular chains. The positive correlation between T_g_ and M_w_ indicates that increasing molecular weight restricts segmental chain mobility, thereby elevating T_g_. This restriction on molecular motion simultaneously reduces deformation under shear creep conditions, thus effectively improving the creep resistance of the material.

The influence of molecular weight on the shear creep behavior of HDPE is primarily mediated by the entanglement density, which can be quantified by the entanglement molecular weight (M_e_). The M_e_ is the average value of the molecular weight between two adjacent entanglement points, which can be calculated from the plateau modulus ($G_{0}^{N}$). As illustrated in Figure 3b, the E’ of the samples decreases with increasing temperature and eventually reaches a stable value. This stabilized plateau region corresponds to the $G_{0}^{N}$, which is used to calculate the entanglement molecular weight. Using Formula (1) [24], the entanglement molecular weights for all samples were calculated, and the results are summarized in Table 2.

(1) $$M_{e}=\frac{\rho RT}{G_{0}^{N}}$$

The M_e_ can reflect the entanglement density of the polymer chain, with the two exhibiting an inverse relationship. A lower M_e_ value indicates a higher entanglement density. Among the five samples, PE32 exhibits the lowest M_e_, indicating the highest entanglement density, whereas PE14 shows the opposite behavior. The higher molecular weight of PE32 promotes the formation of a greater number of entanglement points, thereby resulting in the highest entanglement density among the samples. This enhanced entanglement strengthens intermolecular interactions and constraints, consequently improving the resistance of PE32 to external shear stress. As a result, PE32 exhibits the smallest deformation and superior creep resistance during shear creep tests. In contrast, PE14 demonstrates the poorest creep performance.

**Figure 5:**
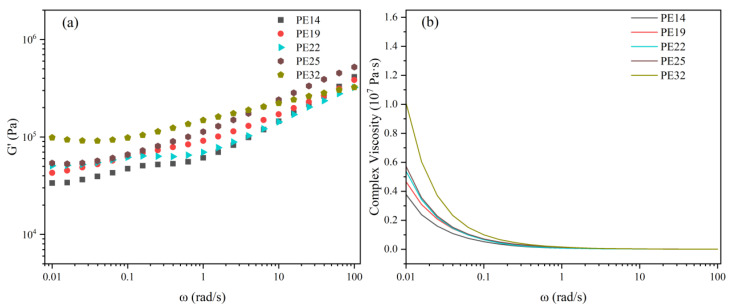
(**a**) The storage moduli G’ and (**b**) the complex viscosity versus frequency curves of PE14, PE19, PE22, PE25 and PE32.

In order to further analyze the mechanism of M_w_ affecting shear creep, rheological tests were conducted on the five samples PE14, PE19, PE22, PE25, and PE32, as shown in Figure 5a,b.

The storage modulus (G’) serves as a key indicator for characterizing the viscoelastic behavior of polymers. Figure 5a shows the variation of G’ with frequency for the five samples. As observed, G’ increases with increasing frequency for all materials. The rise in G’ at high angular frequencies can be attributed to the delayed response of chain segments to rapid external loading [25]. Thus, the low-frequency G’ is more relevant and representative of the material behavior under the shear creep conditions examined in this experiment. At low angular frequencies, PE32 exhibits the highest G’ values, indicating its superior elasticity. This enhanced elasticity, stemming from its high entanglement density, enables PE32 to effectively resist deformation, thereby resulting in its improved creep resistance.

Relaxation time is a fundamental parameter that characterizes the viscoelastic behavior of materials, defined as the duration required for a material to return to its equilibrium state following the removal of an external force that induced deformation. The relaxation time of each sample can be calculated using the Cross formula (Formula (2)) [26]. Figure 5b shows the curves of the composite viscosity of five samples as a function of frequency, and the corresponding relaxation times are 162 s, 180 s, 203 s, 225 s, and 263 s, respectively. Among them, PE14 exhibits the shortest relaxation time, while PE32 has the longest relaxation time. The longer relaxation time can be attributed to the more stable polyethylene chain structure formed by high entanglement density. This more stable structure reduces the creep deformation of HDPE under sustained shear stress and improves the shear creep resistance of PE32.

(2) $$\eta^{*}=\frac{\eta_{0}}{\left[1+{\left(\lambda \omega \right)}^{1-n}\right]}$$

**Figure 6:**
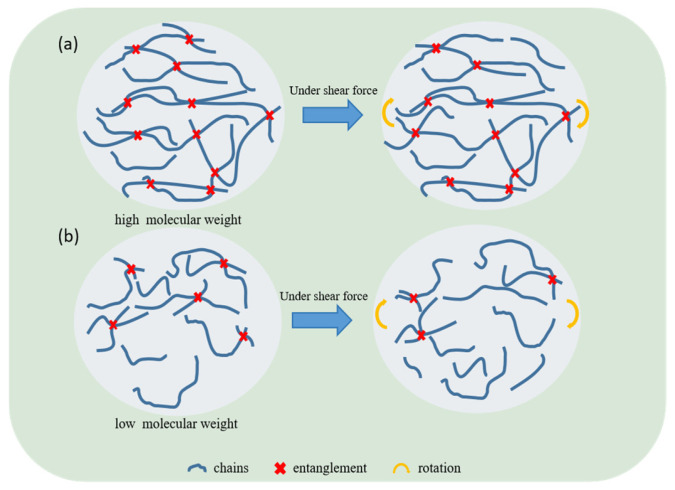
Schematic diagrams of polyethylene shear creep with (**a**) high molecular weight and (**b**) low molecular weight.

Based on the above analysis, it is evident that the M_w_ influences the viscoelastic properties of polyethylene by governing its entanglement density. The specific mechanism is shown in Figure 6. During shear creep, polyethylene deforms primarily through the gradual extension of chain segments and the relative slip between molecular chains. Polyethylenes with higher M_w_ possess longer polymer chains that promote the formation of more entanglement points, leading to a higher entanglement density. This molecular structure with high entanglement density contributes to greater elasticity and substantially improves creep resistance. In contrast, polyethylene with lower M_w_ exhibits reduced entanglement density, which offers less resistance to chain slippage and thus results in inferior creep resistance. These findings demonstrate that under identical shear stress conditions, higher M_w_ polyethylene features higher entanglement density, which effectively restricts chain mobility and thereby strengthens the material’s resistance to shear creep.

### 3.3. Shear Creep Behavior of Polyethylene with Different Comonomer

**Figure 7:**
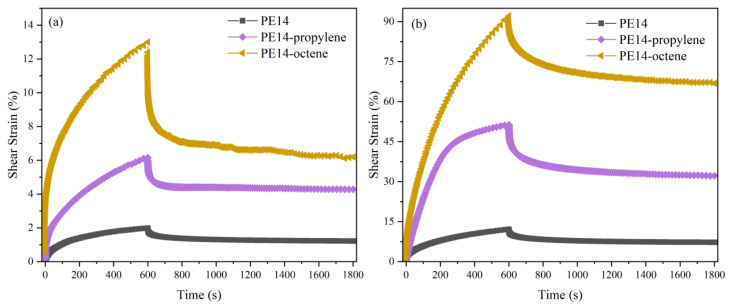
Shear creep and creep recovery curves of PE14, PE14-propylene and PE14-octene under (**a**) 90 °C and (**b**) 210 °C.

In order to determine the effect of different comonomers on the shear creep resistance of polyethylene, three polyethylene samples with similar molecular weight and number of comonomers but different types of comonomers were subjected to shear creep testing. These three samples are ethylene homopolymer PE14, ethylene propylene copolymer PE14-propylene, and ethylene–octene copolymer PE14-octene. The shear creep test results at 90 °C and 210 °C are shown in Figure 7.

During the shear creep process at 90 °C, compared with the initial state, the maximum shear deformation of PE14, PE14-propylene and PE14-octene is 2.0%, 6.2%, and 13.0%, respectively. At 210 °C, the corresponding shear deformation values of PE14, PE14-propylene and PE14-octene are 12.2%, 51.4%, and 92.1%, respectively. This trend can be attributed to the presence of branched chains. The branched chains expand the free volume of polymer molecules, thereby reducing intermolecular interactions. Therefore, in contrast to homopolymer polyethylene, copolymer polyethylene with branched chains is more prone to creep deformation under shear stress and exhibits lower shear creep resistance. Regarding creep recovery, significant permanent deformations still occur in polyethylene with different comonomers after both instantaneous and delayed recovery.

### 3.4. Analysis of Shear Creep Mechanism of Polyethylene with Different Comonomers

**Figure 8:**
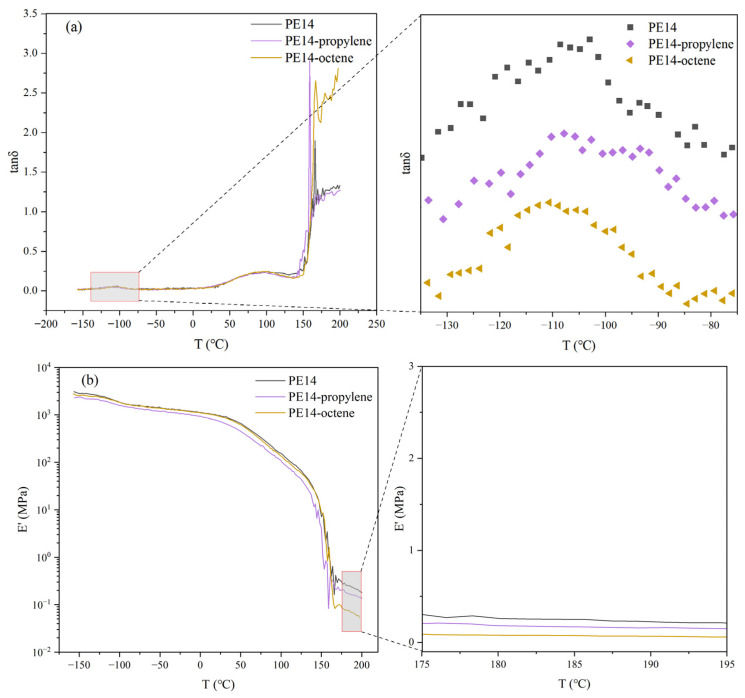
(**a**) The tan δ and (**b**) the storage modulus E’ change-with-temperature curves of PE14, PE14-propylene and PE14-octene.

In order to determine the influence mechanism of different comonomers on the shear creep of polyethylene, DMA tests were conducted on the samples. Figure 8a,b show the variation of the storage modulus E’ and the loss factor tan δ with temperature obtained from temperature scanning of the samples at a fixed frequency.

As mentioned earlier, the shear creep resistance of polyethylene diminishes with decreasing T_g_. Based on the test results shown in Figure 8a, the T_g_ of the three samples is summarized in Table 3. The T_g_ for PE14, PE14-propylene and PE14-octene is −103.0 °C, −107.1 °C and −112.1 °C, respectively. Among the three samples, PE14 exhibits the highest T_g_, whereas PE14-octene shows the lowest. Therefore, PE14 shows the highest creep resistance, whereas PE14-octene exhibits the opposite behavior. This finding is consistent with the results of previous studies on shear creep. The reduction in T_g_ after adding comonomers can be attributed to the introduction of branched chains, which expand the molecular free volume and enhance chain mobility.

As previously discussed, the entanglement density calculated by the $G_{0}^{N}$ serves as an effective parameter for analyzing the influence of comonomers on the shear creep behavior of materials. As shown in Figure 8b, the $G_{0}^{N}$ of PE14, PE14-propylene and PE14-octene is 0.213 MPa, 0.149 MPa, and 0.058 MPa, respectively. The entangled molecular weights of the samples calculated according to Formula (1) are summarized in Table 3. PE14 exhibits the lowest entanglement molecular weight and the highest entanglement density, whereas PE14-octene shows the highest entanglement molecular weight and the lowest entanglement density. This result can be attributed to the branched structure of PE14-octene, which increases the free volume of molecular chains and thereby reduces the entanglement density. Under an applied stress, low entanglement density allows for easier chain slippage and deformation, leading to its poor resistance to shear creep. Due to its high entanglement density, PE14 strengthens the intermolecular forces, effectively restricts segmental motion, and thereby gives rise to a significantly improved shear creep resistance.

**Figure 9:**
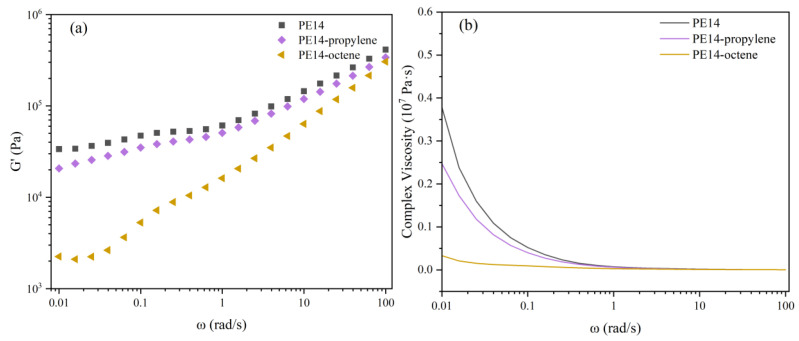
(**a**) The storage modulus G’ and (**b**) the complex viscosity versus frequency curves of PE14, PE14-propylene and PE14-octene.

In order to further analyze the influence mechanism of different comonomers on shear creep, rheological tests were conducted on the three samples—PE14, PE14-propylene, and PE14-octene—from the perspectives of modulus and relaxation time. The results are shown in Figure 9a,b.

Figure 9a shows the storage modulus variation curves of three samples with scanning frequency. The G’ of the three materials decreases with the decline in frequency. At low angular frequencies, PE14 exhibits the highest G’ values, indicating its superior elasticity. In contrast, PE14-octene shows the lowest G’ values, reflecting its poorest elasticity. The decrease in G’ can be attributed to the reduction in chain entanglement density. The lower entanglement density is insufficient to effectively restrict the motion of molecular chains, resulting in greater deformation of the material under shear stress. Consequently, PE14-octene exhibits the poorest resistance to shear creep.

Figure 9b presents the complex viscosity-frequency curves of the three samples. The relaxation time of each sample was calculated from composite viscosity using Formula (2). The relaxation times of PE14, PE14-propylene and PE14-octene are 162 s, 113 s, and 49 s, respectively. PE14 exhibits the longest relaxation time, while PE14-octene shows the shortest. The shorter relaxation time is attributed to a lower entanglement density, which reduces intermolecular interactions and makes the material more prone to deformation. In contrast, PE14 has the longest relaxation time and exhibits a lower deformation tendency, thus having a stronger resistance to shear creep.

**Figure 10:**
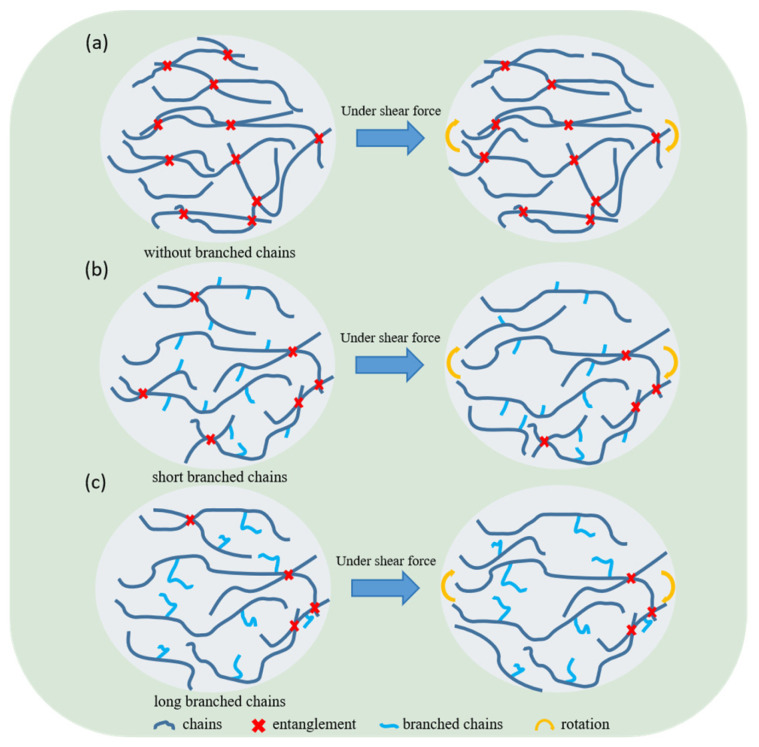
Polyethylene shear creep schematic diagrams (**a**) without branched chains, (**b**) with branched side chains and (**c**) with long branched chains.

Based on the above analysis, the influence of different comonomers on the shear creep of polyethylene is illustrated in Figure 10. Compared to homopolymer polyethylene without branched chains, the introduction of comonomers leads to the formation of branched chains, which increase the free volume of the system. The increased free volume reduces entanglement density and weakens intermolecular interactions. With lower entanglement density, the constraints on molecular chain motion become insufficient, allowing the chains to slide past each other more readily. Consequently, the presence of branched chains diminishes the material’s creep resistance. On the other hand, copolymers prepared with different comonomers exhibit varying branch lengths, which also influence creep resistance. Longer branched chains further expand the free volume, leading to an even lower entanglement density. Under shear stress, a lower entanglement density facilitates easier relative sliding between molecular chains. As a result, polyethylene with longer branched chains exhibits the weakest creep resistance. In summary, the presence of branched chains increases molecular free volume, reduces entanglement density, and weakens interchain interactions, thereby lowering the material’s resistance to shear creep. This detrimental effect becomes more pronounced with increasing branch length.

## 4. Conclusions

This article investigates the effects of average molecular weight and comonomer on the shear creep behavior and underlying mechanisms of HDPE. Based on the comprehensive investigation, it can be concluded that both molecular weight and structural branching play critical roles in governing the material’s resistance to shear creep. The study demonstrates that increasing the weight-average molecular weight significantly enhances creep resistance by promoting greater molecular chain entanglement, which effectively restricts chain mobility under shear stress. Conversely, the introduction of comonomers such as propylene and octene can lead to the formation of branched structures, which expand the free volume within the polymer system, reduce entanglement density, and weaken creep resistance. The longer the branched chains, the more significant the inhibitory effect on entanglement density, leading to a further decline in creep resistance. These findings provide guidance for the development of more creep-resistant polyethylene materials in long-term use environments.

## Figures and Tables

**Table 1:** Technical attributes of polyethylene.

Sample Name	M_n_/g·mol^−1^	M_w_/g·mol^−1^	d	CH_3_/1000C	SCB ^a^/1000C	ρ/g·cm^−3^
PE14	62,100	148,100	2.4			0.9986
PE19	80,000	191,800	2.4	2.9	2.5	0.9937
PE22	92,100	226,500	2.5			0.9936
PE25	109,900	252,700	2.5			0.9939
PE32	144,800	325,100	2.2			0.9948
PE14-propylene	67,700	149,000	2.2	13.0	12.5	0.9986
PE14-octene	54,800	135,000	2.5	11.5	11.0	0.9907

^a^ SCB: short-chain branched.

**Table 2:** Glass transition temperature, plateau modulus, and entanglement molecular weight of PE14, PE19, PE22, PE25 and PE32.

Sample Name	T_g_/°C	$G_{0}^{N}$/MPa	M_e_/g·mol^−1^
PE14	−103.0	0.213	18,234.6
PE19	−102.2	0.289	13,389.1
PE22	−101.3	0.421	9225.2
PE25	−100.7	0.543	7124.2
PE32	−100.1	1.029	3762.8

**Table 3:** Glass transition temperature, plateau modulus, and entanglement molecular weight of PE14, PE14-propylene and PE14-octene.

Sample Name	T_g_/°C	$G_{0}^{N}$ /MPa	M_e_/g·mol^−1^
PE14	−103.0	0.213	18,234.6
PE14-propylene	−107.1	0.149	26,061.1
PE14-octene	−112.1	0.058	66,587.7

## Data Availability

The original contributions presented in this study are included in the article. Further inquiries can be directed to the corresponding author.
